# Denture cleanliness and hygiene: an overview

**DOI:** 10.1038/s41415-022-4397-1

**Published:** 2022-07-08

**Authors:** Petros Mylonas, Paul Milward, Robert McAndrew

**Affiliations:** School of Dentistry, College of Biomedical and Life Sciences, Cardiff University, Heath Park, Cardiff, CF14 4XY, UK

## Abstract

Dentures are an excellent treatment modality for partial and edentate patients; however, improper denture care and hygiene can result in both decreased longevity of the prosthesis and increased risk of developing dental caries, periodontal disease and oral candidosis. Previously, it has been shown that patients and dental professionals are unaware of the different materials and methods available for optimum denture care and hygiene. This article provides an overview of the key legislation and main commercially available methods for denture cleanliness and hygiene, and serves as a basis for providing tailored denture hygiene for denture wearers.

## Key points

- Patients typically demonstrate poor levels of denture hygiene which impacts oral health.
- Denture hygiene instructions comprise mechanical and chemical methods; both should be tailored to the denture wearer.
- Denture base and denture teeth materials had different compatibility with different denture cleaning methods.

## Introduction

Dentures are custom-made medical devices prescribed by dentists and clinical dental technicians to replace oral hard and soft tissue structures. According to an Adult Dental Health Survey, almost one in five adults wear dentures in the UK, with denture wearing increasing with age.^1^Dentures consist of the denture base and denture teeth. Denture bases can be fabricated using acrylic (polymethylmethacrylate [PMMA]), metal alloys (cobalt-chromium) and polymers, such as nylon-based thermoplastic resins, polyether ether ketone and aryl ketone polymer. Heat-cured PMMA is the most commonly used denture base; this is inherently porous, non-shedding and readily aggregates denture plaque. Denture teeth can be fabricated from acrylic, composite resin, or porcelain as these are significantly smoother surfaces; however, denture plaque can grow around the 'tooth-gingivae' interface, which represents the interface between the denture teeth and pink resin of the denture, representing the gingival tissue.^2^^,^^3^^,^^4^

Denture plaque contains pathogenic microbes including, *Candida albicans* (linked with denture stomatitis) and S*treptococcus mutans* (linked with caries development). Methicillin-resistant *Staphylococcus aureus* has been previously isolated from denture patients in general practice and the general hospital setting; however, it is unknown whether cross-contamination or infection can occur when handling dentures.^2^^,^^3^^,^^4^^,^^5^ Most patients exhibit poor denture hygiene due to inadequate knowledge of optimal/correct cleaning techniques^6^^,^^7^^,^^8^ and a lack of standardisation in denture hygiene assessments; dentists infrequently assess and record patients' denture hygiene status.^9^^,^^10^ Poor denture hygiene leads to increased risk of dental caries, periodontal disease, denture stomatitis and halitosis.^6^^,^^11^^,^^12^

Improper denture care negatively impacts denture clinical longevity and increases denture plaque aggregation. For example, scratches due to improper brushing technique, such as brushing with too hard a brush, can increase microbial growth, while inappropriate denture cleaner use can permanently damage a denture beyond clinical use. A working knowledge of denture base/tooth materials and denture cleaning methods should ensure optimal denture cleanliness without compromising material integrity and clinical longevity.^13^^,^^14^^,^^15^ Therefore, clinician-assisted patient education can improve patients' denture hygiene. The dental team is best suited to help provide patients with the necessary oral and denture hygiene education.^9^^,^^10^^,^^16^^,^^17^

This paper provides an overview of the key legislation, current and future methods of denture care and hygiene and compatibility with existing denture materials. It also offers recommendations according to denture material type.

## Current legislation

The Medical Devices Regulations Act 2002,^18^ Medicines and Healthcare Products Regulatory Agency (2013) and the Medical Devices Directive^19^ classify dentures as a Class IIa (custom-made) medical device. They are prosthetic devices which are specifically manufactured for individual patients, intended for long-term continual use in the oral cavity and according to a written prescription by a dental professional. Additional care and maintenance instructions should be provided.

## Assessing denture cleanliness

The Denture Cleanliness Index (DCI) allows simple and rapid evaluation of denture hygiene by semi-quantitatively grading severity according to the amount of staining on the fitting surface of the denture. This can be used by dentists and dental care professionals. DCI scores range from 0 (best) up to 4 (worst) and are designated according to the DCI criteria.^9^^,^^10^

**Fig. 1:**
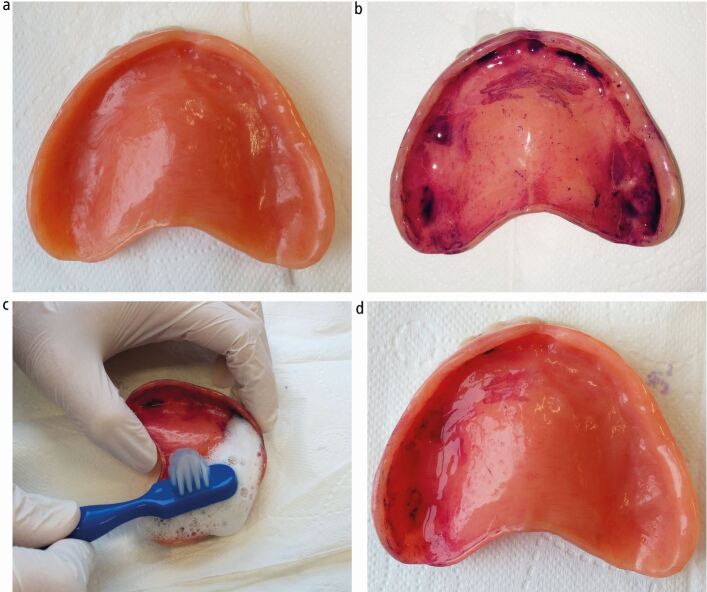
Using the Denture Cleanliness Index and providing denture hygiene instructions. a) Denture gently washed under cold water to remove loose debris. b) Liquid plaque disclosing solution applied to entire denture fit surface, left for 30 seconds, excess washed under cold water. DCI score determined. c) Denture hygiene demonstrated and provided to patient. d) Patient shown cleaned half and asked to clean remaining half. Patient instruction leaflet given

The patients' denture is first gently washed under cold water to remove loose debris before a liquid plaque disclosing dye is then applied onto the entire denture fit surface. After 30 seconds, excess is washed off, the fitting surface visually inspected and DCI score given (see Table 1 and Figure 1). For patients with both maxillary and mandibular dentures, a DCI score is recorded for both and the worst score is the patients' overall DCI score.

**Table 1:** The Denture Cleanliness Index

Score	Description
0	Clean denture. No plaque is visibly seen, no staining, no plaque detectable
1	Denture is visibly clean. Little staining (<25% fit surface stained)
2	Denture has visible plaque and/or debris. Moderate staining of fit surface (25-50% fit surface stained)
3	Denture has visible plaque and/or debris. Severe staining of fit surface (>50% fit surface stained)
4	Denture has visible calculus deposit on any surface
*	Visible defects in denture, in addition to any of the above score (defects defined as those which are potentially plaque retentive or require repair or remake of denture)

Denture hygiene instruction (DHI) are provided according to their DCI scores (Table 2); this process is analogous to oral hygiene instructions (OHI) and recorded in the clinical records accordingly.

**Table 2:** Denture hygiene instructions provided according to DCI score

Score	Strategy
0	No intervention required, reinforce current denture hygiene
1	Denture hygiene reinforcement
2	Denture hygiene reinforcement, patient information leaflet
3	Denture hygiene reinforcement, patient information leaflet and denture hygiene kit
4	Denture hygiene reinforcement, patient information leaflet, denture hygiene kit Intervention by clinician to clean dentures
*	Consider denture reline or remake (depending on severity of defect)

## Current methods of denture hygiene

Two main methods are available: mechanical and chemical and it has been previously recommended that patients use a combination of both to ensure optimal denture plaque removal.^20^

### Mechanical methods

Mechanical methods include use of manual- (using a brush) and/or vibrational-based (using an ultrasonic or sonic bath) cleaning aids.

### Manual cleaning

A regular toothbrush with soap and water have been previously reported as the most common method of denture cleaning.^6^ Proprietary denture brushes and mechanical cleaning adjuncts minimise risk of scratching, which reduces risk of biofilm accumulation and improves clinical longevity of the prostheses. While normal handwashing soap or dishwashing soap has been previously described as a popular method for use together with a denture or toothbrush, they may not provide antimicrobial properties similar to those in specifically formulated chemical denture cleaning agents,^21^^,^^22^ Adjuncts to assist manual cleaning can be divided into pastes, gels, foams, or powders (Table 3); each have similar ingredients with similar modes of actions. These are designed to enhance the cleaning capabilities of normal manual cleaning methods.^6^^,^^7^^,^^21^

**Table 3:** Manual cleaning adjuncts: denture cleaning gels, foams, powders and dentifrices

Manual cleaning adjuncts	Common ingredients	Mode of action	Commercial example(s)
Denture cleaning paste ('denture toothpaste')	Water, hydrated silica (abrasive), sorbitol (sweetener), glycerin (humectant), PEG-6 (dispersant), sodium lauryl sulphate (detergent), carrageenan (stabiliser), saccharin sodium (sweetener), CL 42090 (colourant - blue)	Similar to conventional toothpastes but with much finer silica particles, formulated to minimise scratching	Dentu-Crème Denture Cleaning Toothpaste
Denture cleaning gel	Citric acid, eucalyptus oil, sea salt	Mildly acidic, antibacterial with some abrasive component	Curaprox Daily Gel Cleaner
Denture cleaning foam	Water, glycerin, sesamum indicum (sesame) seed oil, aroma, sorbitol, sodium lauryl sulphate, PEG-40 hydrogenated castor oil, cocamidopropyl betaine, sodium benzoate, PEG-400, benzoic acid, PVM/MA copolymer, sodium saccharin, disodium EDTA, BHT, limonene, linalool	Kills 99.9% of odour-causing bacteria^*^ Low abrasive formula, gentle cleaning action Feel fresh breath for up to five hours	Polident Fresh Cleanse Foaming Cleanser
Denture cleaning powder	Potassium monopersulphate (oxidising agent) sodium lauryl sulphate,sodium bicarbonate, citric acid, flavourants	Oxidising formulation, dislodges debris/biofilm, and antibacterial	Kleenite Denture Cleanser Powder Steradent Denture Cleaning Powder
Key: * = In laboratory studies			

The choice between the use of a denture brush or a regular soft-bristled toothbrush must be discussed with patients on an individual basis, taking into account patient dexterity, ease of use and access for cleaning their respective denture.

### Vibration-based cleaning baths

Vibrational cleaning baths are subdivided according to frequency of vibrations as either ultrasonic or sonic. Vibrational cleaning baths can be used with a bespoke chemical cleaner (manufacturer specific for the bath) or using distilled or tap water.

#### Ultrasonic cleaning bath

Ultrasonic cleaning systems function with a typical frequency range between 20-60 kHz. The vibrational energy causes cavitation; bubbles form, collide and implode with the surface debris, dislodging from the denture surface.^23^^,^^24^ These baths have the best debris removal capability and are compatible with almost all denture material types; however, their high cost may prevent their widespread use by patients,^23^^,^^25^^,^^26^

#### Sonic cleaning bath

Sonic cleaning baths operate a much lower frequency range, usually less than 10 KHz and typically function in 6.5 KHz range. They produce bubbles similarly to ultrasonic baths but are not as effective in cleaning dentures due to the lower vibrational energy imparted by the device. Although these units as significantly more cost effective, there may be cost implications due to the battery usage required.^23^^,^^25^^,^^26^

### Chemical methods

Denture cleaning chemicals are specifically formulated for disinfection of any oral prostheses and must only ever be used extraorally to prevent harm to the patient.^27^

They can be categorised according to their chemistry/mode of action:

- Bleach based, and may contain:
  - Sodium hypochlorite
  - Sodium hydroxide
- Effervescent type, and may contain:
  - Peroxide
  - Bicarbonate
  - Percarbonate
  - Persulphate
- Mineral acid based
- Enzyme based
- Oral rinses
- Flexible denture cleaners.

#### Bleach-based denture cleaners

Bleach-based cleaners contain sodium hypochlorite at 1.5% or 2% w/v and/or sodium hydroxide (1.7% w/v) and have the best and broadest antimicrobial capabilities. The antimicrobial action of sodium hypochlorite is attributed to the hydroxyl (OH^-^) and chloride (Cl^-^) ions dissociated in water, which cause the dissolution of microbial cell walls, dissolution of mucins, degradation of lipids and fatty acids, and irreversible enzymatic inactivation.^28^

They can be used for short duration (typically 10-20 minutes) or overnight disinfection according to manufactures instructions and will vary depending on the dilution ratios specified. A bleach-based denture cleaner consisting of minimum 0.5% hypochlorite solution used for at least three minutes daily was associated with sufficient antimicrobial activity against *Streptococcus mutans* and *Candida albicans*, without changes to acrylic colour, surface roughness or mechanical properties.^29^ Although the main disadvantage of these cleaners is the risk of acrylic discolouration and degradation of metal based components, the risk is dependent on concentration and duration of immersion.^30^^,^^31^

#### Effervescent type denture cleaners

Effervescent tablets consist of oxidants such as sodium bicarbonate, sodium percarbonate and sodium persulphate, that release carbon dioxide bubbles on dissociation in water, while hydrogen peroxide-containing cleaners release oxygen.^32^^,^^33^^,^^34^ Sodium lauryl-sulphate is a commonly added detergent to aid biofilm disruption and improve cleaning efficacy of the oxidants present.^13^ While the antimicrobial activity is inferior compared with bleach-based denture cleaners, effervescent-type denture cleaners can be used for cleaning metallic dentures; there have been no reported instances of corrosion from their use.^35^^,^^36^ Generally, these cleaners should be avoided in dentures modified with a chairside or laboratory-fabricated acrylic reline material. They degrade these lining materials over time, resulting in hardening and increasing porosity of the resilient liner.^23^^,^^34^^,^^37^

#### Mineral acid-based denture cleaners

Mineral acid-based cleaners, typically containing hydrochloric or phosphoric acids, are infrequently used in the UK and are more popular internationally. They chemically dissolve any calcified biofilm deposits and the cell membrane of microorganisms in organic biofilms. Extensive tarnishing and corrosion of metallic components will occur if these are utilised with metal alloy-based dentures and their use is therefore contraindicated.^23^^,^^38^

#### Enzyme-based denture cleaners

Enzyme-based cleaners are seldom used in the UK and their composition is similar to effervescent-type cleaners, with the addition of different enzymes, such as lipases, amylases and proteases. They are formulated to degrade fats, glycoproteins and other proteinaceous organic matter, contributing to their antimicrobial activity. They are primarily used with dentures that have had soft reline materials used; there has been limited evidence to show a negative effect on common denture reline materials.^23^^,^^25^^,^^26^

#### Oral rinses

Oral rinses encompass any oral care product marketed for use as a mouthwash, with examples including 0.2% chlorhexidine gluconate, 0.05% salicylate solution (a derivative of salicylic acid) and phenolic-based mouthwashes, such as Listerine (Johnson and Johnson, USA). Their use as denture cleaners has been widely reported; however, their antimicrobial properties vary widely.

Chlorhexidine-based mouthwashes (at 0.2% concentration) are the most commonly used oral rinse and recommended for use by oncology patients rehabilitated with oral prostheses.^8^^,^^39^ Concentrations between 0.2-4% have been demonstrated to provide significant antimicrobial activity, with 4% solution (five-minute soak) providing superior antimicrobial properties against *Candida albicans* and *Streptococcus mutans* on acrylic dentures and dentures with soft silicone linings compared to mechanical brushing and effervescent-type cleaning tablets.^40^

Prolonged use (daily for several months) of chlorhexidine solutions (0.2-4%) can stain dentures in a fashion similar to natural teeth.^23^^,^^25^^,^^26^^,^^41^ Dentures immersed in 2% chlorhexidine solution resulted in perceptible colour changes (brown-like discolouration) after seven days of continuous usage, when compared to dentures soaked in 0.5% sodium hypochlorite solution for three minutes daily for 90 days.^29^ The use of chlorhexidine as a denture cleaner has been demonstrated to provide good antimicrobial and biofilm removal capabilities; however, to minimise the risk of denture staining, its use should be for limited short periods.

#### Flexible denture cleaners

Flexible dentures are produced thermoplastic polyamide resins, such as nylon and have a limited range of flexible movement. Reported advantages when compared with conventional denture materials (heat-cured PMMA and cobalt-chromium) include increased patient comfort and metal-free construction.^42^ These dentures are produced using manufacturer-specific components with companies supplying their own denture-care recommendations. Additionally, a manufacturer's warranty is provided with these denture types, which may be voided if a denture cleaner other than the one recommended by the manufacturer is utilised.

In general, most flexible dentures are cleaned with a specific silicone-bristled denture brush/toothbrush, together with a specified flexible denture cleaner.^42^ All current flexible denture cleaners have similar formulation and functionality to effervescent-type denture cleaners and typically consist of an oxidant, such as potassium peroxymonopersulphate or potassium peroxydisulphate and acids, such as sodium benzoic acid and citric acid.

## Denture cleaning method compatibility with existing denture materials

Any denture cleaning method can influence the physical and aesthetic characteristics of denture materials if not used according to manufacturer's recommendations. As a result, both patients and clinicians must be aware of the uses and limitations of these methods according to the denture material time to ensure optimal clinical longevity.

While a previous Cochrane systematic review concluded there was a lack of suitable evidence to determine the efficacy of one cleaning method over another,^43^ a recent systematic review determined that bleach-based (sodium hypochlorite) denture cleaners possessed the best and broadest antibacterial and fungicidal activity of all chemical denture cleaners; however, the contact or immersion time required to achieve the required antimicrobial activity was correlated with the solution concentration.^13^ An overview of the compatibility between denture cleaners and denture materials can be seen in Table 4.

**Table 4:** Compatibility of denture cleaning methods with different denture materials

Denture cleaning method	Acrylic dentures	Metal dentures	Dentures modified with soft or resilient linings	Flexible dentures	Polymer-based dentures
Denture brush			X	X	✓
Toothbrush	✓	✓	X	X	✓
Silicone brush	✓	✓	X	✓	✓
Bleach-based	✓	X	✓	X	✓
Effervescent type	✓	✓	X	X	✓
Mineral-acid-based	✓	X	X	X	✓
Enzyme-based	✓	✓	✓	X	✓
Oral rinses	✓	✓	✓	X	✓
Flexible denture cleaner	✓	✓	✓	✓	✓

### Acrylic dentures

Acrylic-based dentures are the most prescribed denture base material and can be easily scratched if bristles are too stiff or made from a material harder than acrylic. Typical denture and toothbrushes are made from nylon where stiffness ranges from soft to medium; these typically will not scratch acrylic. Scratch depths greater or equal to 0.20 μm (micrometres) in depth are required for microbial attachment and scratches increase the surface area for microbial adhesion and biofilm formation.^38^^,^^44^

Ordinary dental toothpaste contains silica particles which act as an abrasive, which increase the rate of scratch formation and contributes to microbial growth; they should therefore be avoided in cleaning acrylic dentures.^23^ However, proprietary denture cleaning paste 'denture toothpaste' are formulated without these particulates to ensure minimal risk of scratch formation.

There is a widely reported variation in compatibility with chemical denture cleaners. Bleach-based cleaners can permanently alter the colour and physical properties of acrylic if used at too high a temperature (>37 ^o^C), too high a concentration (>2% w/v) and for prolonged immersion periods (days and months).^13^^,^^45^^,^^46^ Effervescent-type and enzyme-based cleaners have no reported compatibility issues with acrylic resins. Chlorhexidine digluconate (0.2% solution) has no reported issues of material incompatibility with any denture material or reline material; however, they have been reported to cause staining of acrylics with prolonged use.^41^

### 'Metallic' dentures

Metal-based dentures are at very low-risk of damage from manual cleaning methods, such as denture brushes and adjuncts, such as denture creams. However, bleach-based and acid cleaners are generally contraindicated as they corrode and tarnish commonly used cobalt-chromium and nickel-chromium alloys, regardless of concentration.^14^^,^^38^^,^^47^ Bleach-based cleaners may be used if the metal alloy used is known to be resistant to short-duration exposure to sodium hypochlorite.^23^^,^^26^^,^^38^ Recent evidence suggests that effervescent-type denture cleaners are highly compatible with no measurable/observable issues with common denture framework metal alloys.^48^

### Flexible dentures

The thermoplastic polyamide resins are resistant to many solvents and cleaning agents; however, manufacturers of these resins recommend their own bespoke/branded denture cleaning systems, including a specific denture brush, chemical cleaning agent and more.^49^ Some manufacturers have stated that if other commercially available denture cleaners are used then this may damage the flexible denture and invalidate the manufacturer's warranty.^50^

### Polymer-based dentures

There is very little research on the chemical compatibility of conventional denture cleaning methods and these new polymer denture materials (aryl ketone polymer-based dentures) have been reported by the manufacturer as compatible with all known existing denture care products and methods.^51^ It is recommended to follow the instructions provided by the manufacturer that supplied the polymer material in the construction of the polymer-based denture.

### Resilient denture-reline materials and tissue conditioners

Resilient denture liners can either be plasticised acrylic resin-based (for example, Coe-soft, GC Japan) or silicone elastomer-based (for example, Molloplast-B, Dentax Germany). Plasticised acrylic resin-based resilient liners contain a plasticiser to ensure softness and resilience of the lining material which could be leached out during denture soaking, leading to hardening/cracking. However, silicone-elastomer resilient liners contain polydimethylsiloxane polymers and no plasticisers and can thus maintain their softness for longer when exposed to different chemical denture cleaners.^52^^,^^53^

Plasticised acrylic resin-based resilient liners were previously found to become harder after shorter periods of immersion in both bleach-based and effervescent-type cleaners (after one month) when compared to silicone elastomer-based liners which required three months before significant differences in hardness were measurable.^53^ Silicone elastomer-based liners have been demonstrated as more compatible with both bleach-based and effervescent-type cleaning solutions.^53^^,^^54^^,^^55^ Effervescent-type denture cleaners are contraindicated for use with plasticised acrylic resin reline materials and their impact on the softness and roughness of silicone elastomer-based reline materials is worse when compared with bleach-based cleaners.^53^^,^^54^^,^^55^

Bleach-based denture cleaners may be considered the most compatible chemical denture cleaner for either reline material type when used in an appropriate manner.^41^^,^^56^ However, it is important to always consider manufacturer's instructions before using any cleaning method with resilient liners or tissue conditioners. For example, the instructions for the use of the tissue conditioner Visco-gel (Dentsply, UK) specifically state that commercially available chemical cleansers (all types) are not used and recommend only using gentle cleaning with a soft-bristled brush and clear water.^57^

Therefore, soft mechanical cleaning methods are likely the only commonly accepted method for cleaning any relined denture; however, chemical denture cleaners use must be checked with individual resilient liner manufacturers' recommendations.^52^

## Other methods of denture cleaning and disinfection

Other denture cleaning methods have been reported or are up-and-coming to the commercial market. These methods are worth considering for patients who may not be able to use a mechanical or chemical cleaning method, as described earlier.

### Microwave irradiation

Microwave irradiation use in disinfecting dentures was first suggested in 1985 by Rohrer and Bulard. According to a recent review, this method uses a normal (unmodified) domestic microwave oven to heat a denture immersed in a bowl with tap water; however, there is no agreed nor standardised methodology for their use in cleaning dentures.^58^ Most reported methods immersed dentures under normal tap water before using a microwave oven, with some placing dentures dry in the microwave.^58^ Microwave irradiation has been shown to destroy microorganisms on the surface of denture acrylic, including *Candida albicans* and *Pseudomonas aeruginosa.*^58^ However, the use of microwave irradiation to disinfect dentures remains contentious and should not be recommended over other simpler and more robust cleaning methods.

### Antibacterial denture wipes

These are a new type of denture cleaning method consisting of a wipe impregnated with an antibacterial cleaning solution. They are designed for discreet cleaning of dentures in situations where the usual mechanical and chemical cleaning methods are either impractical or not possible and their use is therefore complementary to the other conventional denture cleaning methods.^59^ Axe *et al.* (2019) determined that denture wipes were well tolerated by participants with no reported issues of oral or dermal reaction and an improvement in both reported quality of life and social confidence.^59^ There have yet to be studies which have assessed the antimicrobial efficacy of these denture wipes compared with standard chemical cleaning methods, such as bleach based or effervescent-type solutions; however, it is known that they provide better antimicrobial activity verses wiping only using, for example, a dry tissue.^59^

## Impact of poor denture hygiene

There is a statistically significant relationship between the level of patients' knowledge of denture care and the quality of patients' denture hygiene. It has been previously reported that, of patients that were not provided with information on denture hygiene and care, only 16% demonstrated good levels of denture care.^60^

In partial denture wearers, there is a statistically significant proportional relationship between quality of denture cleaning and frequency cleaning of remaining teeth, with previous studies indicating 92% of those with poor denture hygiene also had equally poor levels of oral hygiene.^60^ Wearing removable partial dentures does not increase the risk of periodontal diseases, provided that the pre-prosthetic periodontal health was optimal, oral and denture hygiene habits are maintained and meticulous.^61^ In the partially dentate, there is therefore an increased incidence of dental caries and gingival diseases in denture wearers with poor denture and oral hygiene,^62^ while in both complete and partial denture wearers there is an increased risk of denture-related stomatitis. The incidence of denture-related stomatitis is significantly higher in those denture wearers that also demonstrate poor levels of denture hygiene.^63^^,^^64^

Denture-related stomatitis is a chronic multifactorial inflammation of the oral mucosa with multifactorial aetiology, including poor denture hygiene, hyposalivation and poorly adapted prosthesis.^65^ The causative factor in the development of denture-related stomatitis is the presence of *Candida albicans* on the denture surface and oral mucosa in contact with the denture. The clinical impact of denture stomatitis is significant and can negatively impact the quality of life of denture wearers, with both clinical signs (erythema and oedema of the palatal mucosa) and self-reported symptoms (dysgeusia and burning sensation) implicated due to denture stomatitis.^65^ In patients with optimal denture hygiene, the incidence and recurrence of denture stomatitis is significantly reduced.^63^

## Discussion

To reduce the incidence of oral diseases associated with poor denture hygiene, patients must be educated in good denture hygiene/cleaning practice, as it has been widely reported that patients do not receive adequate denture hygiene/care instructions.^12^^,^^15^^,^^66^

The most common mechanical method for cleaning dentures is a toothbrush and ordinary dental toothpaste and the most commonly used chemical cleaning agent were effervescent-type cleaners.^6^^,^^12^^,^^67^^,^^68^ A recent Cochrane review comparing current methods of cleaning dentures was unable to identify the most effective method for denture plaque removal, due to the lack of available literature on the subject.^43^ However, the consensus among the literature would suggest that the concomitant use of mechanical and chemical cleaning methods provides the optimal reduction of denture biofilm.^8^^,^^69^

Careful explanation to patients regarding the use of chemical denture cleaners extraorally must be explained and reinforced as there have been hypersensitivity cases reported where patients have misused these chemicals according to the Food and Drug Authority (FDA) in the United States. The FDA have issued a public health notification advising healthcare professionals and patients on the correct usage of denture cleaning chemicals.^70^

The chemicals with broadest antimicrobial activity are the bleach-based cleaning agents; however, they can tarnish and corrode metallic components on dentures and thus their use should ideally be limited to acrylic dentures and those that have been modified with reline material.^23^^,^^37^ Effervescent-type cleaning agents also exhibit good antimicrobial activity and do not affect metallic components; however, there have been studies indicating their negative impact on denture reline materials.^23^^,^^37^

Clinicians must be aware of methods that are currently available so that optimum and correct advice can be given, as it has been demonstrated that professionally led and delivered educational instructions on correct denture care can positively improve denture are and oral hygiene.^12^^,^^71^

Other methods, such as microwave irradiation and denture wipes, are also available and have been previously reported for cleaning dentures. The use of microwaves is not recommended for use due to high-risk of irreparable denture damage, while denture wipes provide a complimentary and readily available method of cleaning dentures in situations where conventional cleaning methods may not be available.^59^^,^^72^

## Conclusion

Denture wearers tend not to have good denture hygiene. The provision of both oral hygiene and denture hygiene instructions should be carried out on a regular basis to help patients maintain good oral health. The DCI serves as a simple and quick way of assessing quality of patients' denture hygiene and should be incorporated into routine dental examinations. Bleach-based cleaners have the best antimicrobial activity but can corrode and tarnish metal dentures. Effervescent-based cleaners can be used to effectively clean metal dentures but can degrade denture lining materials. It is important to highlight to patients that dentures must be cleaned extraorally to ensure effective cleaning occurs and to reduce the risks of issues from denture cleansers that may have adverse effects on the oral mucosa.
